# Prévalence et facteurs associés à la lombalgie chez les conducteurs de taxi moto à Porto-Novo (Bénin)

**DOI:** 10.11604/pamj.2019.32.107.13477

**Published:** 2019-03-07

**Authors:** Zavier Zomalhèto, Rose Christelle Nayeton Mikponhoué, Armand Wanvoègbe, Ivanovich Adikpéto, Paul Ayélo

**Affiliations:** 1 Service de Rhumatologie, Centre National Hospitalo-Universitaire Hubert Koutoukou Maga, Cotonou, Bénin; 2 Unité de Recherche en Santé au Travail et Environnement, Cotonou, Bénin; 3 Service de Médecine Interne, Centre National Hospitalo-Universitaire Hubert Koutoukou Maga, Cotonou, Bénin

**Keywords:** Prévalence, lombalgie, taxi-moto, facteurs associés, Porto-Novo, Prevalence, low back pain, motorcycle drivers, factors associated, Porto-Novo

## Abstract

**Introduction:**

la lombalgie constitue un problème majeur de santé au travail. Certaines populations de travailleurs semblent beaucoup plus exposées que d’autres. L’objectif de cette étude était de déterminer la prévalence et les facteurs associés à la survenue de la lombalgie chez les conducteurs de taxi moto à Porto-Novo.

**Méthodes:**

étude transversale à visée descriptive et analytique qui avait concerné 270 conducteurs de taxi-moto ayant au moins un an d’ancienneté et consentants. Ces derniers étaient soumis au questionnaire de type nordique adapté à notre contexte et un examen physique du rachis. Les données collectées ont été analysées à l’aide du logiciel Epidata 3.1. et STATA/IC 11.0. Le seuil de significativité était de 5% et les intervalles de confiance calculés à 95%.

**Résultats:**

la prévalence de la lombalgie était de 68,89%. L’âge moyen des conducteurs de taxi-moto était de 42,43 ±11,25 [25-64] ans. La majorité conduisait depuis plus de 5 ans (93,33%). La durée moyenne de conduite par jour était de plus de 8 heures (93,34%) et 68,52% parcouraient plus de 160km par jour. La lombalgie était d’horaire mécanique dans 91,39%. Le mode d’installation était brutal dans 81,73%. La douleur était modérée chez 55,91% des conducteurs, d’évolution aigue (46,24%) et sans irradiation dans 62,36% des cas. L’âge, le niveau d’instruction, le stress, la posture, l’état des amortisseurs de moto étaient associés à la lombalgie chez ces conducteurs de taxi-moto (p < 0,001). Par contre la distance parcourue, la durée de travail, l’ancienneté et l’état des routes n’étaient pas associés à la survenue de la lombalgie.

**Conclusion:**

la lombalgie demeure un sérieux problème de santé publique notamment chez les conducteurs de taxi-moto dans notre pays où la conduite de moto devient de plus en plus un métier de secours pour la population confrontée au chômage grandissant d’où la nécessité d’agir sur les différents facteurs pour une prévention efficace.

## Abstract (en)

**Introduction:**

low back pain is a major health problem in the workplace. Some populations of workers appear to be much more exposed than others. The aim of this study is to determine the prevalence and the factors associated with the occurrence of low back pain among motorcycle drivers in Porto-Novo.

**Methods:**

we conducted a cross-sectional descriptive and analytical study of 270 consenting motorcycle drivers with at least one year of seniority. Patients were asked to complete the Nordic Questionnaire adapted to our context and physical examination of the spine was performed. Data were analyzed using Epidata 3.1. Software and STATA/IC 11.0. The threshold significance level was 5% and the confidence intervals were 95%.

**Results:**

the prevalence of low back pain was 68.89%. The average age of motorcycle drivers was 42.43 ±11.25 [25-64]years. The majority of them had been driving for more than 5 years (93.33%). Drivers spent an average of more than 8 hours (93.34%) driving per day and 68.52% of them drove over 160km per day. Low back pain was mechanical in 91.39% of cases. It was characterized by sudden onset in 81.73% of cases. Pain was moderate in 55.91% of drivers, evolving in acute pain in 46.24% of drivers and without radiation in 62.36% of cases. Age, education level, stress, posture, condition of motorcycle shock absorbers were associated with low back pain in these motorcycle drivers (p < 0.001). By contrast, distance travelled, job duration, seniority and road conditions were not associated with the occurrence of low back pain.

**Conclusion:**

low back pain is a serious public health problem in particular among motorcycle drivers in our country where riding a motorcycle is becoming more and more a work to bring assistance to the population facing growing unemployment. Hence the need to address factors associated with effective prevention.

## Introduction

La prévalence annuelle des lombalgies chez les adultes de 30 à 64 ans dans la population générale est estimée à 54% chez les hommes en France [1]. En Afrique, les études trouvent des fréquences allant de 29,59% à 40% [2-6]. Les lombalgies constituent un motif fréquent de consultation en médecine du travail. Elles peuvent être imputables à une activité professionnelle impliquant des postures ou des mouvements inconfortables de manière répétitive, accompagnées d'efforts relativement importants [7]. Les travailleurs des secteurs du bâtiment et du transport constituent les catégories professionnelles les plus exposées avec des prévalences variant entre 20,5 et 60,4% [8, 9]. En 2014 au Nigéria, une étude réalisée chez les conducteurs de véhicules de transport avait trouvé des prévalences de 50,5% dans l’Etat d’Osun et de 64,8% en 2011 à Ibadan [10, 11]. Au Bénin, le fort taux de chômage a donné naissance depuis quelques années au phénomène de taxi moto, communément appelé «Zémidjan» et leur nombre ne cesse de s’accroitre avec une forte concentration dans les milieux urbains, tels que Porto-Novo, la capitale administrative. La présente étude a pour but d'apprécier la prévalence et les facteurs associés à la survenue des lombalgies chez ces conducteurs de taxi-moto dans la capitale du Bénin.

## Méthodes

Il s’agissait d’une étude transversale, descriptive et analytique qui s’est déroulée de 1^er^ juillet au 31 octobre 2015. La population cible était constituée de conducteurs de taxi-moto répondant aux critères suivants: 1) être conducteur depuis 1 an; 2) exercer dans la ville de Porto-Novo; 3) avoir au moins un an d’ancienneté dans la pratique du métier; 4) être enregistré à la mairie; 5) être consentant. Cette étude a requis l’approbation du comité éthique de la Faculté des Sciences de la Santé du Bénin. Un tirage aléatoire sans remise de 04 arrondissements (constituant 4 grappes) sur les 05 de la ville de Porto-Novo a été réalisé. Les conducteurs de taxi-moto ont été recrutés à partir de leurs points de regroupement dans les arrondissements. Au total, 270 conducteurs avaient été sélectionnés. Ces derniers ont été soumis au questionnaire nordique adapté à notre contexte [12] et à un examen systématique du rachis lombaire. Les conditions de travail: la durée de conduite, le siège de la douleur, son caractère mécanique ou inflammatoire, l’existence d’antécédents lombaires ont été explorés. Les données collectées ont été dépouillées après validation des fiches remplies. Le dépouillement informatique s’est effectué à l’aide du logiciel Epidata 3.1. L’apurement et l’analyse des données ont été faits à l’aide du logiciel statistique STATA/IC 11.0. Les comparaisons ont été faites à l’aide du test de chi2 ou le test exact de Fisher. Le seuil de significativité a été de 5% et les intervalles de confiance ont été calculés à 95%.

## Résultats

### Caractéristiques socio-démographiques des conducteurs

Tous les 270 conducteurs de notre échantillon étaient de sexe masculin. La moyenne d’âge était de 42,43 ±11,25 [25-64] ans. Plus de la moitié avaient un niveau d’instruction primaire. Sur le plan professionnel, la majorité des conducteurs de taxi-moto (93,33%) travaillait depuis plus de 5 ans et avait une masse horaire hebdomadaire supérieure à 40 heures. Le Tableau 1 résume les caractéristiques socio-démographiques et professionnelles des patients.

**Tableau 1:** caractéristiques socio-démographiques des conducteurs

Caractéristiques socio-démographiques	Effectif	Pourcentage
**Age (ans)**		
˂ 30	28	10,37
30 à 40	102	37,78
41 à 50	48	17,78
˃ 50	92	34,07
**Niveau d’instruction**		
Primaire	166	61,48
Secondaire	64	23,70
Non scolarisé	40	14,81
**Situation matrimoniale**		
Mariés monogames	264	97,78
Mariés polygames	06	2,22
**Ancienneté dans le métier**		
3-5ans	18	6,67
˃ 5ans	252	93,33
**Nombre d’heures de travail par jour**		
˂ 8	18	6,66
≥ 8	252	93,34
**Posture adoptée**		
Penchée en avant	130	48,15
Penchée en arrière	13	4,81
Tronc bien dressé	127	47,04
**Le conducteur est couché sur la moto aux heures de repos**		
Oui	55	20,37
Non	215	79,63
**Distance parcourue**		
˂ 160	85	31,48
≥ 160	185	68,52
**Port de bagages**		
Oui	55	20,37
Non	215	79,63
**Lombalgie avant le début du métier**		
Oui	48	25,80
Non	138	74,20
**Traumatisme du rachis**		
Oui	10	3,70
Non	260	96,30

### Caractéristiques cliniques

La prévalence de la lombalgie était 68,89%. Il s’agissait plus fréquemment de douleur de début brutal chez les conducteurs (81,73%), d’intensité modérée (55,91%) et d’horaire mécanique (91,39%). les caractéristiques de la douleur sont résumées dans le Tableau 2. Par ailleurs la déformation du rachis la plus fréquente était l’attitude scoliotique observée chez 55,91% des conducteurs.

**Tableau 2:** caractéristiques cliniques des conducteurs de taxi-moto

Caractéristiques	Effectif	Pourcentage (%)
**Mode de début**		
Brutal	152	81,73
Progressif	34	18,27
**Durée d’évolution**		
Aiguë	86	46,24
Subaiguë	52	27,96
Chronique	46	25,80
**Horaire**		
Mécanique	170	91,39
Inflammatoire	16	8,61
Mixte	00	00
**Irradiation**		
Aucune	116	62,36
Mal systématisé	42	22,58
L3	15	8,07
L4	13	6,99
**Côté atteint**		
Aucun	116	62,36
Droit	55	29,57
Gauche	13	6,99
Droit et gauche	02	1,08
**Signes accompagnateurs**		
Paresthésies	00	00
Troubles génito-sphinctériens	00	00
Claudication intermittente	92	49,46
Aucun	94	50,54
**Intensité selon EVA**		
Modéré	104	55,91
Intense	74	39,78
Sévère	08	4,31
**Déformation du rachis**		
Attitude scoliotique	104	55,91
Scoliose	01	0,54
Hyperlordose	00	00
Aucune	81	43,55
Syndrome rachidien	94	50,54

### Facteurs associés à la survenue de la lombalgie

L’âge > 50 ans, le niveau d’instruction, la posture figée, penchée en avant pendant la conduite et le mauvais état des amortisseurs étaient associé à la survenue des lombalgies chez les conducteurs (p<0.001). Ces facteurs associés sont résumés dans le Tableau 3.

**Tableau 3:** facteurs associés à la lombalgie chez les conducteurs de taxi-moto

Facteurs	Lombalgie		RC	P
	Oui	Non		
**Age**				
˂ 30	13(46,43%)	15(53,57%)	1,35[0,58-3,12]	
30-40	55(53,92%)	47(46,08%)	1	p ˂ 0,001
40-50	08(16,37%)	40(83,33%)	5,85[2,49-13,73]	
˃ 50	08(8,70%)	84(91,30%)	12,28[5,39-27,98]	
**Niveau d’instruction**				
Primaire	78(46,99%)	88(53,01%)	1	
Secondaire	02(3,13%)	62(96,88%)	7,47[6,5-116,05]	p ˂ 0,001
Non scolarisé	04(10,00%)	36(90,00%)	7,97[2,71-23,42]	
**Posture**				
Penchée en avant	17(43,08%)	67(47,86%)	1	p ˂ 0,001
Tronc bien dressé	113(86,92%)	73(52,14%)	0,16 [0,08-0,30]	
**Etat des amortisseurs**				
Très bon	05(55,56%)	04(44,44%)	1	
Bon	77(39,09%)	120(60,91%)	1,94[0,5-7,48]	p ˂ 0,001
Mauvais	02(3,13%)	62(96,87%)	38,75[5,64-266,03]	
**Satisfaction au travail**				
Non	65(37,36%)	109(46,43%)	1	p ˂ 0,003
Oui	19(19,79%)	77(80,21%)	2,41[1,34-4,35]	
**Stress au travail**				
Non	15(93,73%)	01(6,25%)	1	p ˂ 0,001
Oui	69(27,17%)	185(72,83%)	40,21[5,21-310,23]	
**Modèle de Karasek adapté**				
Travail stressant	36(47,57%)	43(54,43%)	1	
Travail dynamique	47(24,74%)	143(75,26%)	2,54[1,46-4,42]	p ˂ 0,001
Travail passif	01(100%)	00(00,00%)		
**IMC**				
Normal	49(40,50%)	72(59,50%)	1	p ˂ 0,003
surpoids	35(23,49%)	114(76,51%)	2,21[1,31-3,74]	

## Discussion

L’âge moyen des « zémidjans » était de 42,43 ±11,25 ans (25 à 64 ans). Ndiaye M *et al.* en 2007 au Sénégal rapportaient des données similaires, 40 ± 8 ans (extrêmes de 24 et 57 ans) dans une étude portant sur le personnel d’une entreprise publique de transport [13]. La prévalence de la lombalgie était de 68,89% dans notre étude. Contrairement aux études de Ojo *et al.* [10], Akinpelu *et al.* [11] au Nigéria dont les prévalences sont proches des nôtres, nos résultats sont supérieurs aux 23% rapportés par Ndiaye *et al.* [13] dans une étude similaire au Sénégal Cette différence pourrait s’expliquer par le mode de transport. La lombalgie survient donc chez des sujets relativement jeunes et en pleine activité professionnelle, ce qui pourrait être un handicap à l’exercice de leur profession. Cette hypothèse avait déjà été émise par Vuillaume dans son ouvrage sur « la lombalgie commune: données épidémiologiques et questions de santé publique » [14]. Par ailleurs, dans notre série, chez la plupart des sujets lombalgiques, les symptômes sont survenus des années après le début des activités ce qui suppose un effet cumulatif des facteurs de risque liés au métier de conducteur. De plus, classiquement, les conducteurs de taxi-moto de notre série travaillaient en moyenne 48 heures par semaine; ce qui est bien au-dessus des 40h conseillés par semaine [15].

L’influence de l’âge s’expliquerait par une diminution dans le temps des capacités d’adaptation et de régénérescence du noyau des disques intervertébraux [16]. L’analphabétisme est statistiquement associé à la survenue de la lombalgie (p < 0,001). Le faible niveau d’instruction et la nécessité de subvenir aux besoins de la famille pourrait expliquer le surmenage au travail qui ne peut être qu’un facteur aggravant de la lombalgie. De même, le faible niveau d’instruction est à l’occasion d’une méconnaissance des mesures d’hygiène du dos expliquant le fait pour ces conducteurs d’adopter des postures contraignantes allant jusqu’à se coucher sur leurs motos aux heures de repos.

La posture « penché en avant » pendant la conduite était statistiquement associée à la survenue de la lombalgie (p < 0,001). Van Nieuwenhuyse *et al.* en 2006 avaient objectivé un risque accru de lombalgie chez les travailleurs adoptant une posture statique figée [17]. Les auteurs évoquent la position assise prolongée, les flexions et rotations fréquentes du tronc au poste de conduite combinées aux vibrations du corps entier comme étant susceptibles d’être à l’origine des lombalgies chez des chauffeurs de bus.

L’effet de la mauvaise posture sur la survenue des lombalgies est accentué par un mauvais état des amortisseurs. Ce dernier, combiné au mauvais état des routes empruntées engendreraient des vibrations importantes du corps entier pendant la conduite. La littérature démontre une association entre la lombalgie et l’exposition aux vibrations globales du corps [16]. Chaque cycle de vibration entraîne des étirements et tassements successifs des disques intervertébraux; il en résulterait des modifications des caractéristiques élastiques des disques, suivies de lésions [17].

Par ailleurs, le stress au travail était statistiquement associé à la survenue de la lombalgie (p < 0,001). De nombreuses études ont démontré le rôle des facteurs psychosociaux dans la survenue des lombalgies [18,19]. Ndiaye *et al.* avaient trouvé une association statistiquement significative entre la lombalgie et le stress (p = 0,004) [13]. De même Miyamoto *et al.* avaient identifié le stress professionnel et l'insatisfaction au travail comme étant des facteurs de risques de lombalgie chez les conducteurs de taxi à Tokyo [8]. Le surpoids était statistiquement associé à la lombalgie dans notre étude (p = 0,003). Roquelaure *et al.* avaient également démontré une association positive entre la lombalgie et obésité chez leurs employés [7]. Ceci pourrait s’expliquer par une augmentation de la charge supportée par les disques intervertébraux.

## Conclusion

La lombalgie professionnelle pose un véritable problème de santé publique. Au terme de cette étude transversale à visée descriptive et analytique, il en ressort que la lombalgie est très fréquente chez les conducteurs de taxi-moto de Porto-Novo et que son origine est multifactorielle: facteurs biomécaniques (posture, vibrations..), facteurs psycho-sociaux (stress).

### Etat des connaissances actuelles sur le sujet

- La lombalgie professionnelle pose un véritable problème de santé publique et est source d’une perte socio-économique considérable notamment pour les entreprises;
- Le taux élevé de chômage est à l’origine de nombreuses professions émergentes notamment dans le domaine de la conduite; ainsi la conduite de moto comme moyen de transport est très répandue en Afrique de l’Ouest notamment au Bénin;
- La prévalence de la lombalgie est mieux connue avec les conducteurs de voitures que chez les conducteurs de motos.

### Contribution de notre étude à la connaissance

- Cette étude révèle la particularité que constitue le métier de conducteurs de moto;
- Cette étude révèle le danger que constitue à long terme ce métier notamment sur la qualité de vie des conducteurs: ce métier doit constituer un tremplin et non un métier définitif;
- Cette étude a permis d’identifier certains facteurs associés à la survenue des lombalgies chez ces conducteurs: des mesures peuvent donc être prises pour limiter la survenue des lombalgies chez ces sujets.

## Conflits d’intérêts

Les auteurs ne déclarent aucun conflit d’intérêts.
